# The professor I knew

**DOI:** 10.4103/0972-9941.30687

**Published:** 2007

**Authors:** N D Motashaw

**Affiliations:** Consultant Gynaecologist and Obstetrician, P D Hinduja National Hospital and Breach Candy Hospital, Mumbai - 400016, India

Have we all not heard the phrase “Be present on a particular day, at a particular place, at a particular time to achieve something you desire.” My association with the late Dr. Kurt Semm began in such a manner.

Professor Kurt Semm was visiting King Edward Memorial Hospital on a working day with his paraphernalia. For the first time laparoscopy was being demonstrated in Mumbai. A crowd of residents, anaethetists and students surrounded him. I was outside the theatre looking through the square glass at the VIPs inside and the Professor talking in his loud, clear voice. Suddenly he saw me and said “Why do you not want to be in the theatre and see the pelvic structure?” Room was made for me by everybody and it was a beautiful surgical sight “A sight for the Gods” I exclaimed. Professor then showed me the liver, the small bowel, the big bowel, the rectum, and the abdominal cavity. That was the start of our friendship.

Before leaving the hospital he gave me his card. He was stationed in Munich and worked with Prof. Richard Fickenster. About a year later I visited Munich for a week; he demonstrated a variety of normal pelvic and other pathological conditions.

A couple of years later I visited Dr. Hans Lindermann at Hamburg. He is the pioneer of hysteroscopic surgery. I remember the day when Prof. Kurt Semm was very angry with me for I refused his invitation to Kiel saying it was bitterly cold in Kiel and I could not visit him but visited Dr. Lindermann instead. Prof. Mettler who was working with Prof. Semm still remembers the episode.

A few years later he was taking a group of Germans to Japan for an endoscopy meeting. On the way he spent a couple of days in Mumbai. It was fortunately a Sunday. Every few minutes the telephone bell wound ring for Prof. Semm and he would come back to me and my sister and say – “Can two more people have lunch over here?” This went on repeatedly until the house was full of people, all of us eating and drinking away. The Professor was very clever. Every few minutes he would go inside the kitchen, open all the dish tops and put his index finger in the boiling pot or pan and after tasting come out and in a child-like manner say “It was very tasty.”

This was his first visit to our home; subsequently he always came home whenever he visited Mumbai - he would be so tired from lecturing that he would go to the guest room and sleep.

He possessed profound knowledge not only in “endoscopy” but in instrumentation, jewellery, tourism, sailing and aviation. At almost every visit to Kiel he would take us up in his plane. It was great fun!

On one of his last visits I informed him that some of my instruments needed attention. So each and every instrument was opened up, separated, cleaned and put right by him. On one such occasion my sister pleaded with him to pack his bags or he would miss his plane – he said “No stops, no fears.”

In the car he told the chauffer to speed through the city while we were seated at the back. He the checked in and ran onto the tarmac blowing kisses towards us.

He later suffered from Parkinson's disease and passed away at the age of 76 years. I lost a wonderful friend, so did many Indians who knew him.

